# Breastfeeding Practices among Native Hawaiians and Pacific Islanders

**DOI:** 10.1155/2016/2489021

**Published:** 2016-09-28

**Authors:** Ingrid K. Richards Adams, Chizimuzo T. C. Okoli, Akilah Dulin Keita, Ana Maria Linares, Keiko Tanaka, Joshua R. Polanin, Annie Koempel

**Affiliations:** ^1^Department of Dietetics and Human Nutrition, University of Kentucky, 118 Funkhouser Building, Lexington, KY 40506, USA; ^2^University of Kentucky, 315 CON Building, Lexington, KY 40536, USA; ^3^Institute for Community Health Promotion, Department of Behavioral and Social Sciences, Brown University, Box G-S121-8, Providence, RI 02908, USA; ^4^University of Kentucky, Lexington, KY, USA; ^5^Peabody Research Institute, Vanderbilt University, Nashville, TN, USA; ^6^Department of Dietetics and Human Nutrition, University of Kentucky, Lexington, KY, USA

## Abstract

*Background*. Breastfeeding is associated with a decreased risk of obesity in the early and adult years. Native Hawaiians and Pacific Islanders (NHPI) experience high rates of obesity which is often obfuscated with aggregated data. Using disaggregated data, we examined breastfeeding practices among NHPI.* Methods*. Seven databases and reference lists were searched. Two independent researchers extracted relevant studies based on predetermined criteria. Nine studies met our inclusion criteria and a meta-analysis was conducted using random-effects, inverse-various weighted models.* Results*. Few studies disaggregated NHPI populations when examining breastfeeding practices. Most studies were cross-sectional and our search yielded no randomized or quasirandomized control trials. The results of the meta-analysis indicated that 46.5% NHPI women initiated breastfeeding with 40.8% breastfeeding exclusively. These pooled analyses show that NHPI breastfeeding practices are below the recommended national and international goals and guidelines.* Conclusion*. Breastfeeding practices among NHPI are heterogeneous and critical disparities exist among certain NHPI subgroups and additional research needs to be conducted to determine the reasons for the disparity. Future studies should work to disaggregate data for NHPI and the various subpopulations. Multicomponent, multilevel strategies are needed to support breastfeeding practices among NHPI.

## 1. Introduction

Obesity prevention begins with breastfeeding [1] and infancy (0 to 3 years) is a critical period in obesity development [2]. Recent research suggests a 15% to 30% reduction in adolescent and adult obesity rates with breastfeeding during infancy compared with none [1, 3–7]. Breastfeeding's effects are dose dependent, with exclusive breastfeeding (EBF) and breastfeeding for long durations offering increased benefits [4, 8–13], including several short- and long-term medical, neurodevelopmental, and immunological cognitive advantages [14–19]. Due to these benefits, EBF is recommended for a minimum of six months after birth, followed by continued breastfeeding for a minimum of one year [1, 20–23].

Native Hawaiian and Pacific Islanders (NHPI) are a rapidly growing population and constitute approximately 1.2 million of the Asian American population either alone (44%) or in combination with other races (56%) [24]. Because NHPI are often conflated with Asian Americans and other Pacific Islanders in national and state-level data, disparities among them are often unnoticed. For example, when breastfeeding data are disaggregated by race and ethnicity, studies show that NHPI have lower breastfeeding initiation and EBF rates and have shorter breastfeeding duration than other populations [25–27]. This is concerning because NHPI populations experience higher rates of obesity and obesity-related comorbidities and mortality than other populations, but improving breastfeeding practices can ameliorate obesity-related issues [28–34]. Although steps have been taken to separate Asian and NHPI in census data, the problem of aggregating these groups is still prevalent [35].

Currently, there are no systematic reviews on breastfeeding practices that disaggregate data on NHPI. The information from such a systematic review can provide policy makers, researchers, and public health workers a means to support and enhance breastfeeding practices among NHPI while addressing the problem of high obesity. Therefore, the objectives of this study are to provide a systematic review and meta-analysis of breastfeeding practices used by NHPI in terms of initiation, EBF, and duration.

## 2. Methods

Searches were conducted in July 2013, October 2014, and updated in January 2016 in the online bibliographic databases PubMed, AGRICOLA, CENTRAL, CINAHL, PsychInfo, Sociological Abstracts, and Web of Knowledge for studies examining breastfeeding practices among NHPI. We used Medical Subject Heading (MeSH) terms in PubMed and other indexing terms for the respective databases, as well as text wording. Our initial searches combined the following terms:* breast feeding, breast-feeding, breastfeeding, breastfed, breast fed, breast-fed, child, preschool, infant*,* Pacific Island, Oceanic ancestry group, Hawaii, breastfeed, bottle feeding, formula fed, formula milk, human milk, infant feeding, weaning, child preschool, infant, and pregnancy*. We did not include language or date restrictions in our searches. Two researchers (*IA and AK*) independently scanned the reference lists of all relevant papers retrieved and extracted relevant studies using predetermined inclusion and exclusion criteria. Discrepancies were resolved through discussion so that consensus could be attained.

### 2.1. Inclusion

Studies were included if data were disaggregated for NHPI populations and if they were directly related to breastfeeding practices. Excluded articles were those that were conducted outside of the United States or the Pacific Islands (Micronesia, Melanesia, and Polynesia) or qualitative in nature.

### 2.2. Coding

Two researchers independently coded studies for type, sample size, outcome measures, and effect size. The effect size of interest was the proportion of mothers who adopted certain breastfeeding practices (initiation, exclusive breastfeeding, and duration) or children on whom these practices were used.

### 2.3. Synthesis

We performed two synthesis techniques: (1) a narrative report of breastfeeding practices among NHPI samples in all studies and (2) a meta-analysis of various breastfeeding practice measures using a random-effects, inverse-various weighted model, with effect sizes grouped according to similar measurement constructs. For example, we synthesized measures of breastfeeding initiation separately from breastfeeding duration and exclusivity. Heterogeneity among the effect sizes was calculated using *I* ^2^ index [43]. All analyses were conducted using R package* metafor* [44].

## 3. Systematic Review

**Figure 1:**
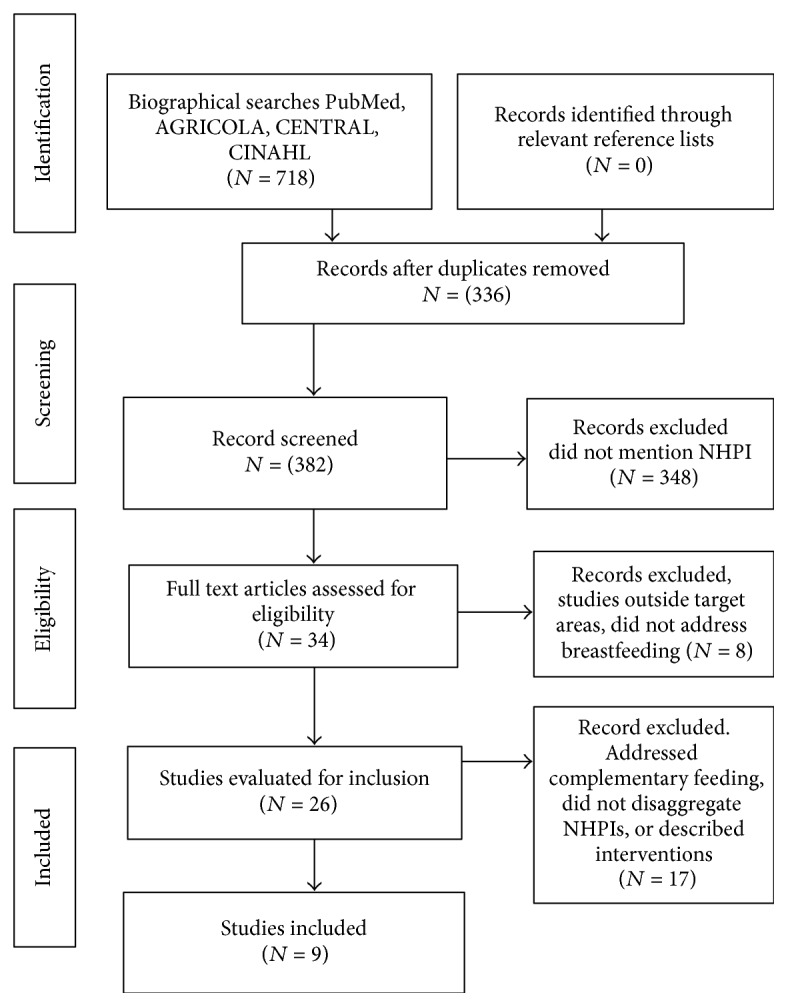
Prisma flow diagram of selected studies.

We identified 718 articles from which we removed 336 duplicates; and, of 382 articles screened for potential relevance, 348 were excluded. Thirty-four articles were assessed for eligibility. Further studies were excluded because they (1) were outside of the United States or Pacific Islands (*N* = 8), (2) addressed complementary feeding or did not disaggregate NHPI from other ethnic groups (*N* = 16), (3) described interventions (without reporting effects) (*N* = 1), and were secondary data from other included studies. Hence, nine studies were included in our final results. See Figure 1.

## 4. Main Findings of the Selected Studies

Study dates ranged from 1969 to 2013. Six studies were conducted before the year 2000. Studies were cross-sectional [26, 27, 36, 37, 39, 41] and each one was cohort [38], panel [40], and univariate descriptive [42]. Furthermore, just over half of the studies were conducted in Hawaii or used Hawaiian datasets [26, 27, 36, 39, 41], and six focused on ethnically diverse populations [26, 27, 36, 39–41], four only on populations in the Pacific Islands [37, 38, 40, 42], and one solely on Native Hawaiians [27]. See Table 1.

## 5. Breastfeeding Initiation

Four studies examined breastfeeding initiation, three among Hawaiians and one in the Pacific Islands. In the first study, an initiation rate of 29.0% for Hawaiian mothers was observed as compared to 26.0% Caucasians and 50.0% Japanese [36]. In the second study, a 52.6% breastfeeding rate at hospital discharge was observed for Native Hawaiian women as compared to 70.4% Caucasian, 55.4% Japanese, and 33.3% Filipino [39]. In the third study, a 1-month initiation rate of 91.0% was observed for Native Hawaiian mothers attending WIC clinics [27]. Finally, among Pacific Islanders, all mothers on Honiara and Nggela-Sandfly breastfed their children [37]. The results of the meta-analysis indicated that less than half of mothers initiated breastfeeding (M = 46.5%, 95% CI (26.9–66.1), *I* ^2^ = 95.22).

## 6. Exclusive Breastfeeding

Six studies reported on EBF [26, 27, 38, 40–42] with three in Hawaii and three in the Pacific Islands. The first study among only Native Hawaiians, reported 3- and 6-month EBF rates of 29.0% and 18.0%, respectively [27]. The second study showed a 31.8% EBF rate among Hawaiian mothers as compared to 51.9% White, 38.0% Korean, 37.0% Black, 33.8% Chinese, 29.4% Japanese, 28.9.4% Filipino, and 24.2% Samoan [26]. The third study found an 18.4% EBF rate among Hawaiians but did not disaggregate the data by ethnicity [41]. The fourth study in Fiji reported EBF rates of 68.5% at 3 months [40]. However, a decline was shown from 60.2% in 1977 to 45.6% in 1980. The fifth study in Kiribati reported EBF rates of 92.0% at 4 months [38]. The final study in the Commonwealth of the Northern Mariana Islands also found a decline in EBF rates with 46.0%, 23.0%, 26.0%, and 9.0% of children exclusively breastfed for 0–2 months, 2–4 months, 4–6 months, and 9 or more months, respectively [42]. Meta-analysis results showed that less than half of mothers reported EBF practices (M = 40.8%, 95% CI (17.8–63.7), *I* ^2^ = 99.74).

## 7. Breastfeeding Duration

Four studies in our review measured breastfeeding duration, three among Pacific Islanders and one among Native Hawaiians. The first study observed that 45.7% of all infants in Kiribati were breastfed at 12 months [38]. The second study among mothers in the Commonwealth of the Northern Marianas Island reported that 22.0% received breastmilk for 12 or more months [42]. The third study [37] mentioned that most mothers in both Honiara and Nggela-Sandfly were breastfeeding at 6 and 12 months. The fourth study in Oahu [27] reported a 6-month breastfeeding duration rate of 52.0% for all mothers. Due to the variability in measures of breastfeeding duration a meta-analysis could not be appropriately performed.

## 8. Discussion

The high incidence of obesity, low breastfeeding rates, and other chronic diseases among NHPI makes it imperative to study differences in breastfeeding practices in these populations. Our review found that breastfeeding practices among NHPI are heterogeneous, highlighting disparities in breastfeeding practices among certain NHPI subgroups [26]. Examining breastfeeding practices among NHPI can expose hidden health issues and trends, making it possible to unearth disparity [45].

Our systematic review yielded only nine studies spanning a 44-year period. Moreover, we found limited disaggregated data in national surveys and surveillance systems for these groups. We found no studies examining breastfeeding patterns and disaggregating data for NHPI in the US where approximately two-thirds of NHPI reside [34]. The NHPI population is a young population that is expected to increase exponentially by the year 2060 [46, 47], and it is also critical that mandates related to disaggregating NHPI data by distinct ethnic and racial groups separate from Asian Americans are implemented [34, 46]. There is a need to enhance the monitoring of breastfeeding practices in all US NHPI states, territories, and commonwealths to better identify risk factors for poor breastfeeding outcomes.

In addition, we found that varying terms and time periods were used for breastfeeding initiation, exclusivity, and duration among studies in our review. Such variations have also been observed among federally funded datasets, indicating a need for standardization of breastfeeding outcomes [45]. Future studies should implement consistent terminology for the purpose of comparing effects and replicating studies. In addition, it is important that studies use time frames (breastfeeding at 6 and 12 months and exclusive breastfeeding at 3 and 6 months) that are aligned with national objectives and national data collection and monitoring systems.

The result of our meta-analysis indicated that less than half of NHPI women initiated breastfeeding or breastfed exclusively. These pooled analyses show that NHPI breastfeeding practices are below the* Healthy People* 2020 goals. Qualitative studies have highlighted several factors that may affect breastfeeding practices among NHPI, namely, lack of knowledge, lactation problems, poor family and social support, social norms, embarrassment related to breastfeeding in public, employment and child care issues, and the education of health care providers, so that they can fully support the breastfeeding efforts of NHPI mothers [35, 48, 49]. Further research needs to be conducted to determine the reasons for the disparity in breastfeeding practices. We suggest, at a minimum, disaggregating survey data by the race/ethnicity in order that future analyst can use such data. Our search produced neither randomized nor quasirandomized controlled trials related to breastfeeding among NHPI. Studies are also needed to systematically explore the types of interventions that can enhance exclusive breastfeeding rates and duration among NHPI.

There are few important considerations that need to be noted in interpreting the findings of this review. The unclear definitions of breastfeeding terms, variability in the measurement periods, and the small number of studies are limitations. However, despite these concerns, the need for this study cannot be overemphasized as it illuminates disparities within the NHPI populations and brings to light the needs of a group that could potentially remain on the outskirts of policy decisions and focus.

## 9. Conclusion

Future studies should work to disaggregate NHPI data and identify barriers to breastfeeding among NHPI. Moreover, multicomponent, multilevel strategies directed toward NHPI communities are needed to support breastfeeding practices. Such studies can support existing culturally appropriate practices, advance health promotion activities, and provide directions for policies to minimize health-related morbidity, mortality, and disparities among the NHPI population.

## Figures and Tables

**Table 1:** Description of selected studies.

Authors, year	Type of study	Sample by location/region and demographics	Measures (breastfeeding initiation, duration, and exclusive)	Results (main outcomes and/or effect sizes)
Brown and Adelson, 1969 [36]	Cross-sectional study	Honolulu, Hawaii (mothers and children) Ethnically and socioeconomically diverse Caucasian, Hawaiian, Japanese, and other populations; Low and middle income; Age (mean): children = 2-3 years; Low income mothers = 28.4 years; Middle income mothers = 29 years; *N* children = 281 *N* mothers = 249	*Indicators: initiation and duration* Initiation: ever breastfed Duration: breast fed for at least 6 months	Approximately one-quarter of the women breastfed their infant. More middle than low income mothers breastfed Ever breastfed: 25.0% of all infants (26.0% Caucasians; 50.0% Japanese; 29.0% Hawaiian; 32.0% other populations) Breastfed at 6 months: 80.0% all infants

Jansen, 1979 [37]	Cross-sectional study	Honiara and Nggela-Sandfly, Solomon Islands, Pacific Islands (mothers) Ethnicity: Pacific Islanders Age (mean): Honiara = 28.3 years; Ngella-Sandfly = 30.7 years *N* = 189 (Honiara = 100, Ngella-Sandfly = 89)	*Indicators: initiation and duration* Initiation: ever breastfed Duration: breastfed for at least 6 months; breastfed for at least 12 months	All mothers from Honiara and Nggela-Sandfly breastfed their infant on the first day after delivery. Early weaning before 6 months was uncommon Ever breastfed: 99.5% Breastfed for at least 6 months: Honiara = 85.7%; Nggela-Sandfly = 93.3% Breastfed for at least 12 months: Honiara = 78.6%; Nggela-Sandfly = 92.3%

Franks and Jurgensen, 1985 [38]	Cohort study	Kiribati, Abemama Atoll Village Clinic (children) Ethnicity: Pacific Islanders *N* = 50	*Indicators: exclusive breastfeeding and duration* Exclusive breastfeeding: exclusive breastfeeding for 4 months Duration: breastfed for 12 months	Most children were breastfed for the whole of their first 4 months of life. Just under half were still being breastfed at 12 months Exclusive breastfeeding for 4 months = 92.0% Breastfed for 12 months = 45.7%

Suganuma et al., 1988 [39]	Cross-sectional study	Oahu, Hawaii (mothers) Ethnically diverse: Caucasian = 37.9%; Hawaiian = 20.3%; Filipino = 10.1%; Japanese = 13.2%; other populations = 18.3% Age = not reported *N* = 562	*Indicator: exclusive breastfeeding* Breastfed only at hospital discharge	Over half of the total sample reported exclusively breastfeeding their infant at hospital discharge. Infant feeding pattern varied considerably by ethnic group. The incidence of exclusive breastfeeding at hospital discharge was the highest among Caucasians and lowest among Filipinos. Just over half of Hawaiians breastfed Breastfed only at hospital discharge: total = 57.5%; Caucasian = 70.4%; Hawaiian = 52.6%; Filipino = 33.3%; Japanese = 55.4%; other populations = 51.5%

Lambert and Yee, 1981 [40]	Panel study	Suva, Fiji (infants) Ethnically diverse: Fijian, Indian, and other populations Age = 0–18 months *N* = varied depending on year of study	*Indicator: exclusive breastfeeding* Breastfed only for at least 0–3 months; breastfed only for at least 4–6 months	The extent of breastfeeding declined over the period 1977–1980 among Fijians as well as other ethnic groups Breastfed only for at least 0–3 months: Fijian 68.5%; Indian 33.8%; other populations 43.7% Breastfed only for at 4–6 months: Fijian 48.4%; Indian 26.2%; other populations 19.3%

Kieffer et al., 1997 [41]	Cross-sectional study (utilizing The Nutrition Survey)	Hawaii (mothers) Ethnically diverse: Filipinos US born (2.8%); Filipinos Philippines born (3.8%); Hawaiian (18.4%); Japanese (14.4%); Other/mixed populations (19.2%); White (41.3%). Age = 27–30 years *N* = 2013	*Indicator: exclusive breastfeeding* Breastfeeding only at hospital discharge	Mothers who breastfed were more likely to be white, older, married, and educated, have chosen their feeding method prior to pregnancy, and were primarily concerned with the health of their infant as a reason to breastfeed Exclusive breastfeeding among Hawaiians = 18.4% Data not disaggregated by ethnicity

Dodgson et al., 2007 [27]	Cross-sectional study utilizing WIC data	Oahu, Hawaii Ethnicity: Native Hawaiians Age (mean) = 26.71 years *N* = 200	*Indicator: exclusive breastfeeding and duration* Exclusive breastfeeding: any breastfeeding at 1 month; any breastfeeding at 6 months Duration: exclusively breastfed for at least 3 months; exclusive breastfeeding for at least 6 months	A small amount of women breastfed their infant exclusively for 6 months. Mothers who breastfed exclusively at initiation were significantly more likely to breastfeed for 6 months than women who partially breastfed at initiation. Most Native Hawaiian women combined exclusive breastfeeding and partial breastfeeding. More multiparous than primiparous women exclusively breastfed at initiation Any breastfeeding at 1 month = 91.0%; any breastfeeding at 6 months = 52.0%; exclusively breastfed at least 3 months = 29.0%; exclusive breastfeeding at least 6 months = 18.0%

Novotny et al., 2007 [42]	Univariate descriptive study	Commonwealth of the Northern Mariana Islands (children) Ethnicity: Pacific Islanders Age = 6 months to 10 years *N* = 420	*Indicators: exclusive breastfeeding and duration* Exclusive breastfeeding: Exclusive breastfeeding for 0–2; 2–4; 4–6; and 6 or more months Duration of breastfeeding: breastfed for 9 to 12 months; received breastmilk for 12 or more months	Over three-quarters of the children were breastfed, while just over half were breastfed at 6 months. A smaller percentage was still being breastfed at one year Exclusively breastfed: 46.0% were exclusively breastfed for 0–2 months; 23.0% were exclusively breastfed for 2–4 months; 21.0% were exclusively breastfed for 4–6 months; 9.0% were exclusively breastfed for 6 or more months; 15.0% were breastfed for 9–12 months; 22.0% received breastmilk for 12 or more months

Hayes et al., 2014 [26]	Cross-sectional study by PRAMS data	Hawaii (mothers) Ethnically diverse: Caucasian = 21.3%; Native Hawaiian = 27.6%; Samoan = 2.9%; Filipino = 18.3%; Japanese = 12.2%; Chinese = 3.6%; Korean = 1.6%; Black = 2.4%; other populations = 10.2% Age = 20–35 years *N* = 8082	*Indicator: exclusive breastfeeding* Exclusive breastfeeding at 8 weeks	There was a difference in the rates of exclusive breastfeeding by ethnic group. White mothers had the highest estimate followed by Korean and Black mothers. Disparity in exclusive breastfeeding rates was observed among NHPI subgroups 36.3% of all mothers exclusively breastfed for at least 8 weeks (Caucasian = 51.9%; Native Hawaiian = 31.8%; Samoan = 24.2%; Filipino = 28.9%; Japanese = 29.4%; Chinese = 33.8%; Korean = 38.0%; Black = 37.0%; Other = 41.7%)

## References

[1] American Academy of Pediatrics (2012). Breastfeeding and the use of human milk. *Pediatrics*.

[2] Hawley N. L., Johnson W., Nu'Usolia O., McGarvey S. T. (2014). The contribution of feeding mode to obesogenic growth trajectories in American Samoan infants. *Pediatric Obesity*.

[3] Bergmann K. E., Bergmann R. L., Von Kries R. (2003). Early determinants of childhood overweight and adiposity in a birth cohort study: role of breast-feeding. *International Journal of Obesity and Related Metabolic Disorders*.

[4] Harder T., Bergmann R., Kallischnigg G., Plagemann A. (2005). Duration of breastfeeding and risk of overweight: a meta-analysis. *American Journal of Epidemiology*.

[5] Ip S., Chung M., Raman G., Trikalinos T. A., Lau J. (2009). A summary of the agency for healthcare research and quality's evidence report on breastfeeding in developed countries. *Breastfeeding Medicine*.

[6] Kelishadi R., Farajian S. (2014). The protective effects of breastfeeding on chronic non-communicable diseases in adulthood: a review of evidence. *Advanced Biomedical Research*.

[7] Metzger M. W., McDade T. W. (2010). Breastfeeding as obesity prevention in the United States: a sibling difference model. *American Journal of Human Biology*.

[8] Owen C. G., Martin R. M., Whincup P. H., Smith G. D., Cook D. G. (2005). Effect of infant feeding on the risk of obesity across the life course: a quantitative review of published evidence. *Pediatrics*.

[9] Liese A. D., Hirsch T., Von Mutius E., Keil U., Leupold W., Weiland S. K. (2001). Inverse association of overweight and breast feeding in 9 to 10-y-old children in Germany. *International Journal of Obesity*.

[10] Von Kries R., Koletzko B., Sauerwald T. (1999). Breast feeding and obesity: cross sectional study. *British Medical Journal*.

[11] Hediger M. L., Overpeck M. D., Kuczmarski R. J., Ruan W. J. (2001). Association between infant breastfeeding and overweight in young children. *The Journal of the American Medical Association*.

[12] Armstrong J., Reilly J. J. (2002). Breastfeeding and lowering the risk of childhood obesity. *The Lancet*.

[13] Neyzi G., Binyildiz P., Gunox H., Borms K. (1984). Influence of feeding pattern in early infancy on ponderal index and relative weight. *Human Growth and Development*.

[14] (2012). Breastfeeding and the use of human milk. *Pediatrics*.

[15] Hörnell A., Lagström H., Lande B., Thorsdottir I. (2013). Breastfeeding, introduction of other foods and effects on health: a systematic literature review for the 5th Nordic Nutrition Recommendations. *Food & Nutrition Research*.

[16] Kramer M. S., Aboud F., Mironova E. (2008). Breastfeeding and child cognitive development: new evidence from a large randomized trial. *Archives of General Psychiatry*.

[17] Quigley M. A., Hockley C., Carson C., Kelly Y., Renfrew M. J., Sacker A. (2012). Breastfeeding is associated with improved child cognitive development: A Population-Based Cohort Study. *Journal of Pediatrics*.

[18] Belfort M. B., Rifas-Shiman S. L., Kleinman K. P. (2013). Infant feeding and childhood cognition at ages 3 and 7 years: effects of breastfeeding duration and exclusivity. *JAMA Pediatrics*.

[19] Frederiksen B., Kroehl M., Lamb M. (2013). Infant exposures and development of Type 1 Diabetes Mellitus: the diabetes autoimmunity study in the young (DAISY). *JAMA Pediatrics*.

[20] Breastfeeding http://www.wpro.who.int/mediacentre/factsheets/nutrition_breastfeeding/en/.

[21] Nutrition Exclusive Breastfeeding http://www.who.int/nutrition/topics/exclusive_breastfeeding/en/.

[22] United States Breastfeeding Committee http://www.usbreastfeeding.org/.

[23] Healthy People 2020. https://www.healthypeople.gov/2020/topics-objectives/topic/maternal-infant-and-child-health/objectives.

[25] PRAMS Data on Breastfeeding http://www.cdc.gov/prams/data-breastfeeding.htm.

[26] Hayes D. K., Mitchell K. M., Donohoe-Mather C., Zaha R. L., Melcher C., Fuddy L. J. (2014). Predictors of exclusive breastfeeding at least 8 weeks among asian and native Hawaiian or other Pacific Islander race subgroups in Hawaii, 2004–2008. *Maternal and Child Health Journal*.

[27] Dodgson J. E., Codier E., Kaiwi P., Oneha M. F. M., Pagano I. (2007). Breastfeeding patterns in a community of native Hawaiian mothers participating in WIC. *Family and Community Health*.

[28] McCubbin L. D., Antonio M. (2012). Discrimination and obesity among native Hawaiians. *Hawai'i Journal of Medicine & Public Health*.

[29] Hawaii Physical Activity and Nutrition Plan, 2013–2020. http://health.hawaii.gov/physical-activity-nutrition/files/2013/08/Hawaii-PAN-Plan-2013-2020.pdf.

[30] Global Health Observatory Data Repository: Obesity Data by Country http://apps.who.int/gho/data/node.main.A900A?lang=en.

[31] Ogden C. L., Carroll M. D., Kit B. K., Flegal K. M. (2014). Prevalence of childhood and adult obesity in the United States, 2011-2012. *The Journal of the American Medical Association*.

[32] Baruffi G., Hardy C. J., Waslien C. I., Uyehara S. J., Krupitsky D. (2004). Ethnic differences in the prevalence of overweight among young children in Hawaii. *Journal of the American Dietetic Association*.

[33] Novotny R., Oshiro C. E. S., Wilkens L. R. (2013). Prevalence of childhood obesity among young multiethnic children from a health maintenance organization in Hawaii. *Childhood Obesity*.

[34] http://minorityhealth.hhs.gov/omh/browse.aspx?lvl=3&lvlid=65.

[35] http://empoweredpi.org/wp-content/uploads/2014/06/A_Community_of_Contrasts_NHPI_US_2014-1.pdf.

[36] Brown M. L., Adelson S. F. (1969). Infant feeding practices among low and middle income families in Honolulu. *Tropical and Geographical Medicine*.

[37] Jansen A. (1979). Malnutrition and child-feeding practices among Solomon Islanders in Honiara and Nggela-Sandfly. *The Journal of Tropical Pediatrics and Environmental Child Health*.

[38] Franks A. J., Jurgensen C. (1985). Nutrition and health in the first year of life on a Pacific atoll. Observations on Abemama Atoll, Central Pacific. *Transactions of the Royal Society of Tropical Medicine and Hygiene*.

[39] Suganuma E. K., Alexander G. R., Baruffi G., Gilden S. R. (1988). Infant feeding practices in Hawaii. *Hawaii Medical Journal*.

[40] Lambert J., Yee V. (1981). Suva infant feeding survey. *Fiji Medical Journal*.

[41] Kieffer E. C., Novotny R., Welch K. B., Mor J. M., Thiele M. (1997). Health practitioners should consider parity when counseling mothers on decisions about infant feeding methods. *Journal of the American Dietetic Association*.

[42] Novotny R., Coleman P., Tenorio L. (2007). Breastfeeding is associated with lower body mass index among children of the commonwealth of the Northern Mariana Islands. *Journal of the American Dietetic Association*.

[43] Higgins J. P. T., Thompson S. G. (2002). Quantifying heterogeneity in a meta-analysis. *Statistics in Medicine*.

[44] Viechtbauer W. (2010). Conducting meta-analyses in R with the metafor. *Journal of Statistical Software*.

[45] Chapman D. J., Pérez-Escamilla R. (2009). US national breastfeeding monitoring and surveillance: current status and recommendations. *Journal of Human Lactation*.

[46] http://www.advancingjustice.org/.

[47] Hixson L., Hepler B., Myoung K. (2012). *The Native Hawaiian and Other Pacific Islander Population: 2010*.

[48] Centers for Disease Control and Prevention (2013). *Strategies to Prevent Obesity and Other Chronic Diseases: The CDC Guide to Strategies to Support Breastfeeding Mothers and Babies*.

[49] Oneha M., Dodgson J. (2009). Community influences on breastfeeding described by Native Hawaiian mothers. *Pimatisiwin*.

